# Research quality in scoliosis conservative treatment: state of the art

**DOI:** 10.1186/s13013-015-0046-7

**Published:** 2015-07-11

**Authors:** Fabio Zaina, Michele Romano, Patrick Knott, Jean Claude de Mauroy, Theodoros B. Grivas, Tomasz Kotwicki, Toru Maruyama, Joseph O’Brien, Manuel Rigo, Stefano Negrini

**Affiliations:** ISICO (Italian Scientific Spine Institute), Via Roberto Bellarmino 13/1 20141, Milan, Italy; Italian Scoliosi Study Group (GSS), Vigevano, Italy; Rosalind Franklin Hospital, Chicago, IL USA; Clinique du Park, Lyon, France; “Tsaneio” General Hospital of Piraeus, Piraeus, Greece; Department of Pediatric Orthopaedics, University of Medical Sciences, Poznan, Poland; Department of Orthopaedic Surgery, Saitama Medical Centre, Saitama Medical University, Kawagoe, Saitama Japan; National Scoliosis Foundation, Boston, USA; Quera Salvà Institut, Barcelona, Spain; Deparment of Clinical and Experimental Sciences, University of Brescia, Brescia, Italy; IRCCS Fondazione Don Gnocchi, Milan, Italy

## Abstract

The publication of research in the field of conservative treatment of scoliosis is increasing after a long period of progressive decline. In 2014, three high quality and scientifically sound papers gave new strength to the conservative scoliosis approach. The efficacy of treatment over observation was demonstrated by two RCTs for bracing, and one for scoliosis-specific exercises provided by a physical therapist. It is difficult to design strong studies in this field due to the long time needed for follow up and the challenge of recruiting patients and families willing to be involved in the decision process. Nevertheless, the main methodological errors are not related to the study design but rather on the way it is performed, which very frequently affects the reliability of results. The most common errors are: selection bias, with many studies including functional rather than a true structural scoliosis; inappropriate outcome measures, utilizing parameters not related to scoliosis progression or quality of life; inappropriate follow up, reporting only immediate results and not addressing end of growth results; an incorrect interpretation of findings, with an overestimation of results; and missing the evaluation of skeletal maturity, without which results cannot be considered stable. Being aware of these errors is extremely important both for authors and for readers in order to avoid questionable practices based on inconclusive studies that could harm patients.

## Introduction

Research in the field of conservative treatment of scoliosis is increasing in frequency after a long period of progressive decline lasting from the 1980s to the early 2000s [1–3]. This increase in published studies is a very positive sign for our field. In fact, patients and families have never stopped asking for good conservative treatments [4], and have also asked critical questions about treatment rather than simply accepting the physician’s decision [5, 6]. The combination of patient need and an upward trend in published papers provides a good opportunity to increase the number of researchers in this field, as well as to involve a new generation of physiatrists, together with orthopedic surgeons, in the care of patients with scoliosis.

This is especially important since orthopedists are increasingly engaged in surgical training and performance, and less of their attention goes to conservative treatment [7]. Nevertheless, the quality of research remains a significant issue for every scoliosis treatment. The year 2014 will be remembered for three published Randomized Clinical Trials (RCTs) which were milestones in demonstrating the efficacy of conservative scoliosis treatment. But there are also signs of concern, with some published research studies that we want to identify and address in this paper.

## In 2014 three RCTs gave new strength to conservative scoliosis treatment

For years we have waited for a Randomized Clinical Trial (RCT) to finally validate the effectiveness of brace treatment for scoliosis, with the evidence up until now based only on prospective observational studies [8, 9]. After a first failed attempt [5], a partially randomized trial with a large observational arm showed scoliosis bracing effective in preventing scoliosis surgery, with only 3 patients needed to prevent one surgery [6]. More recently, another RCT comparing bracing and observation has been published, even though it has a small sample population and includes low magnitude curves [10]. It is evident from these two papers that in the field of conservative scoliosis treatment, patients and families want to decide what to do about their therapy. They do not accept the chance of being randomized to the no-treatment arm, and this makes RCTs almost impossible to perform [5, 6]. Moreover, there is now a serious ethical problem with such research. The recruitment of patients in these two RCTs was stopped by their ethical committees because of the dramatic effect of treatment over observation, and this in the end makes it unethical to propose new RCT studies [6, 10]. But there are different study designs that can be adopted, and if performed correctly they can be at least as reliable as RCTs. Well designed prospective controlled studies have demonstrated that they are consistent in their findings with RCTs, even in very high magnitude curves [11], with the advantage of being more economical, more generalizable, and less affected by an overestimation of the effect size [12].

Although bracing is now supported with the new RCTs, significant doubts still exist about another pillar of conservative scoliosis treatment: Exercise Therapy. Although many systematic reviews have concluded that exercises for scoliosis treatment are effective [13, 14], a recent Cochrane review stated that there is lack of high quality evidence in favor of this [15]. Only one of the papers included in the Cochrane review was a RCT, however, many clinical and methodological limitations were detected [12], thus lowering its level of evidence. Nevertheless, the dogma of exercise being ineffective for scoliosis is based on a myth and not science, since no data showing exercise to be ineffective has been published either [16]. “No evidence” doesn’t mean no efficacy; it only means an absence of studies. But now more studies are beginning to be published. Recently, a randomized trial focused on the main principle of modern scoliosis-specific exercise treatment, which is active self correction, showed this movement to be fundamental to obtain the best results [17–19]. That paper confirmed the recommendations of the International guidelines for conservative scoliosis treatment [20], and those of a previous consensus about scoliosis-specific physical therapy exercises, which identified the importance of the principle of self correction [21]. So, the level of research about scoliosis-specific exercise (SSE) provided by a PT is increasing, and hopefully will eventually demonstrate which approaches are most effective [22].

## Common mistakes in scoliosis research

There are some methodological points that must be raised in the attempt for new effective conservative treatments able to reduce the burden of therapies on young patients [9, 20]. In fact, while sound research will convince even the skeptics, biased studies can damage the entire field of conservative scoliosis treatment. Physicians and allied health professionals in the field want to shift their treatment to provide the most effective ones possible, but biased research does not allow this evolution. Therefore, there are some criteria that should be followed in order to collect reliable and useful data.

### Inclusion criteria

Idiopathic scoliosis is not only a matter of posture, but it’s a three dimensional deformity of the spine and trunk [20]. The criteria to define scoliosis requires a coronal curve larger than 10° Cobb Angle, the presence of vertebral rotation in the radiograph and the presence of a rib hump consistent with the curve (measured through the Angle of Trunk Rotation – ATR, or Angle of Trunk Inclination - ATI) [1, 20, 23]. If postural scoliosis [24, 25] (that, in fact, is not scoliosis) is confused with true structural scoliosis, then treatments are proposed for healthy children and good “results” are obviously achieved. Some studies clearly report that they are dealing with postural scoliosis, but a less sophisticated reader can be confused [24, 26]. Also, other studies include curves less than 10° [27, 28], or fail to report data about the ATR [29], and this can be misleading to the reader too.

Other possible limitations of the interpretation of treatment result rely on the inclusion of a mixed population [27, 29]: chronological criteria for scoliosis classification should be followed, and patients should be divided according to the age at diagnosis and scoliosis type (idiopathic, secondary, congenital, degenerative).

### Outcome measures

The main outcome measures for scoliosis have been clearly pointed out [20, 30]. They consist of quality of life assessment, aesthetics, radiographic parameters, ATR/rib hump measurement, and sagittal profile. Papers not reporting these outcomes and claiming improvement of scoliosis cannot be used as pragmatic and appropriate references.

### Inappropriate follow-up

A serious approach to scoliosis during growth must show its effectiveness in the medium term and not only in the very short term. If the results are very short term [27, 29, 31], then the study must be called “preliminary” or “pilot”. No conclusions about final results can be drawn from studies with such a short follow-up other than the indication to go on with further research. One month or 3 month results are useless to reach reliable conclusions on efficacy and 6–12 months of treatment can only show a trend that must be then confirmed with end growth results and possibly a further follow up during adulthood.

### Interpretation of findings

Conclusions must be supported by the data. This sounds obvious, but frequently in all fields of medicine, and also in scoliosis research, there is an overestimation of findings, with statements of effectiveness going beyond what the data actually show [25, 27]. Moreover, if the methods are not sound enough to gather any good evidence, the conclusions are sometimes totally misleading. And this is the real problem; claiming results that have not been proven.

### Skeletal maturity

Scoliosis progression during growth depends on the stage of bone maturation. Despite its limits, the Risser sign is today the gold standard for such an evaluation, and must always be reported in every paper [32]. This sign is important for inclusion criteria and for appropriate follow up. The most risky period for AIS is the growth spurt of puberty, during which the Risser sign changes from 0 to 2. The SRS criteria for bracing studies focused mainly on this phase [33], and skeletal maturity must be reached before the end of treatment to determine the success of the treatment [34].

## The impact of a renewed attention to conservative treatment application and research

In the end, we do not help patients if we cause confusion in the field of conservative treatment.. In fact, during the years bracing was discredited (now apparently finished due to sound and appropriate research), how many patients were treated with the so-called “wait and see approach” that in the end meant only waiting for surgery? This is even more striking when it is recognized that the new epidemiological data indicate that 58 % of patients between 20° and 40° will progress to surgery, or above 50° of Cobb Angle, if left untreated [6]. Now, the scientific challenge in the world of conservative scoliosis treatment is focusing on scoliosis-specific physical therapy exercises. Biased research in this field has the same potential to damage the best studies [15], including a recent high quality RCT [17, 18] and the previous one already existing [35].

## Conclusion

The presence of methodological mistakes in research publications are a problem very well known in the scientific world. Literature based on such mistakes can be very misleading for the general population, since it is not easy for the consumer without expertise to understand what they can trust [36]. Specifically in the field of scoliosis, improper practice based on marketing (through the internet or social media) and not on science, is common, and it is a major reason for concern.

Active scientific groups like the Italian Scoliosis Study Group (GSS) and the international Society on Scoliosis Orthopedic and Rehabilitation Treatment (SOSORT) are working hard to produce and disseminate sound research [20]. Serious evidence about conservative scoliosis treatment is growing in volume, giving physicians more guidelines and tools to help patients. Nevertheless, despite some very good quality papers presenting strong and convincing research, attention is paid to low quality papers that draw the focus of the public away from real science. This low quality published research must be a major concern of the scientific scoliosis community to avoid improper practice, promote professional integrity, and most importantly, to protect patients.
